# Bilateral Optic Neuropathy Secondary to Intravenous Carboplatin Therapy

**DOI:** 10.18502/jovr.v19i1.15448

**Published:** 2024-03-14

**Authors:** Arash Maleki, Mollie K. Lagrew, Elizabeth D. Slaney

**Affiliations:** ^1^Department of Ophthalmology, University of Florida, Gainesville, FL, USA; ^2^The Ocular Immunology and Uveitis Foundation, Waltham, MA, USA; ^3^College of Medicine, University of Florida, Gainesville, FL, USA

**Keywords:** Carboplatin, Chemotherapy-induced Optic Neuropathy, Disc Edema, Optic Neuropathy, Papilledema

## Abstract

**Purpose:**

To report a case of carboplatin-induced bilateral optic neuropathy in a patient with metastatic squamous cell carcinoma of the tongue.

**Case Report:**

A 65-year-old man with a history of squamous cell carcinoma of the tongue with metastasis to the right axillary lymph node treated with carboplatin and paclitaxel was evaluated for decreased visual acuity in both eyes. Visual acuity was 20/70 in the right eye and no light perception in the left eye. On dilated fundus examination, optic disc edema was present in both eyes with more severity in the left eye, flame shape hemorrhages around the optic nerve head in both eyes and cotton wool spots around the left optic nerve head. Brain and orbital MRI demonstrated enhancement of the bilateral optic nerve sheaths. He was diagnosed with bilateral carboplatin-induced optic neuropathy.

**Conclusion:**

Our findings in this case justify monitoring of patients during their course of intravenous carboplatin therapy.

##  INTRODUCTION

Carboplatin belongs to a group of medications known as alkylating agents. It is primarily employed in the treatment of head and neck, lung, breast, gastrointestinal, and ovarian cancers. It may cause maculopathy and cortical blindness weeks after intravenous administration.^[1,2]^ Intracarotid administration can cause severe ocular and orbital toxicity secondary to inflammation.^[2]^ We present a case of carboplatin-induced bilateral optic neuropathy in a patient with metastatic squamous cell carcinoma of the tongue.

##  CASE REPORT

**Figure 1:**
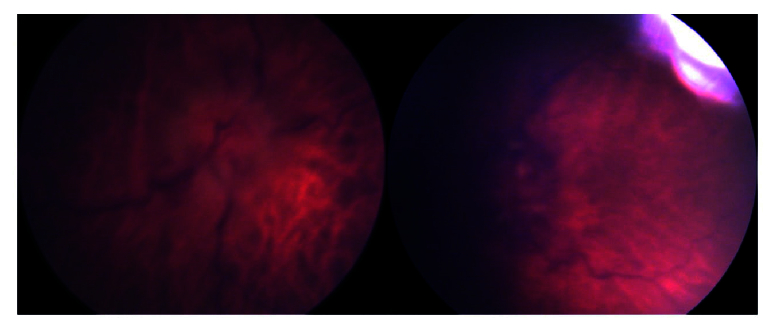
The RetCam images of optic nerve head in both eyes which demonstrates disc edema in both eyes, left (left image) more than right (right image) and flame-shape hemorrhages around optic nerve head in both eyes and cotton wool spots around the left optic nerve head.

**Figure 2:**
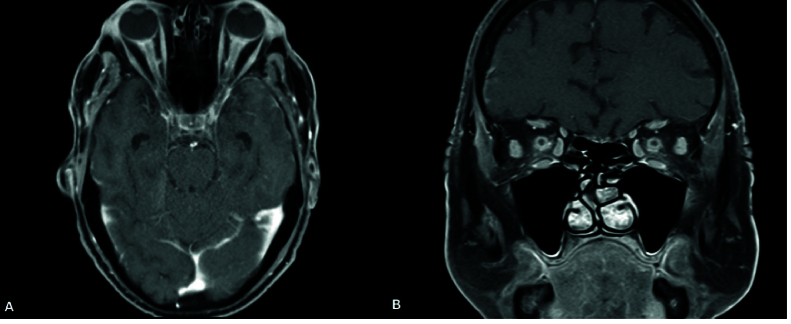
Brain MR, axial view (A) and coronal view (B), demonstrates enhancement of the bilateral optic nerve sheaths.

A 65-year-old man with a history of squamous cell carcinoma of the tongue with metastasis to the right axillary lymph node treated with carboplatin and paclitaxel was evaluated for decreased visual acuity in both eyes. The dose of carboplatin was 300 mg/m ${\;}^{2}$ every four weeks and the dose of paclitaxel was 135 mg/m ${\;}^{2}$ every three weeks for a total of three doses. The progressive decline in vision began around four days after his fifth round of carboplatin therapy. The last dose of paclitaxel had been received 11 weeks before the changes in his vision. Visual acuity was 20/70 in the right eye and no light perception in the left eye. On dilated fundus examination, disc edema was visible in both eyes with more severity in the left eye, flame-shape hemorrhages were observed around the optic nerve head in both eyes and cotton wool spots around the left optic nerve head [Figure 1]. Dilation of retinal vessels around the optic nerve head was present. Choroid examination was normal. There were no signs of retinitis, retinal vasculitis, metastasis to the choroid, and paraneoplastic syndromes, including leopard pattern or bilateral diffuse uveal melanocytic proliferation. Opening pressure on lumbar puncture (LP) was normal. Additionally, LP was negative for malignant cells, but it was positive for primary inflammatory markers (high protein, albumin, and IgG index). The brain and orbital magnetic resonance imaging (MRI) demonstrated enhancement of the bilateral optic nerve sheaths [Figure 2]. Complete work-up was negative for syphilis, Lyme disease, bartonella, sarcoidosis, toxoplasmosis, and tuberculosis. All inflammatory markers including anti-neuromyelitis optica (NMO) antibody, anti-myelin oligodandrocyte glycoprotein (MOG) antibody, antinuclear antibody (ANA), anti-double-strand deoxyribonucleic (dsDNA) antibody, antineutrophil cytoplasmic antibody (ANCA) were negative; however, erythrocyte sedimentation rate (ESR) and c-reactive protein (CRP) were 41 mm/h and 28 mg/L respectively. Left temporal artery biopsy (TAB) was negative. A diagnosis of bilateral carboplatin-induced optic neuropathy was made and treatment was started with the administration of 1000 mg methylprednisolone infusion daily for five days. On the sixth day, since there was no improvement in his left eye vision, he received an intravitreal triamcinolone injection and was started on systemic prednisone of 50 mg daily. Upon discharge from hospital five days after intravitreal injection, there was no improvement in his left eye vision; the right eye was stable.

##  DISCUSSION

The literature includes a few cases of optic neuropathy secondary to carboplatin.^[3,4,5]^ The first case of optic nerve involvement was described by Caraceni et al in 1997.^[3]^ The patient was a 52-year-old female diagnosed with ovarian cancer who developed bilateral optic neuropathy with disc swelling during treatment with systemic cisplatin and carboplatin. The disc swelling disappeared in three weeks, leaving bilateral temporal pallor. At the second day of presentation, visual acuity was determined as light perception in both eyes which improved to 10/10 in one year; however, dynamic visual field was abnormal in both eyes.^[3]^

In 2009, Fischer et al reported a patient with carboplatin-induced bilateral papilledema causing partially reversible visual impairment.^[4]^ Chemotherapy consisted of a three weekly intravenous administration of a regular area under curve- (AUC-) determined carboplatin dose. Ocular symptoms started eight days after the fourth round of intravenous carboplatin. The optic nerve head was prominent in the right eye more than the left eye. The brain MRI and diagnostic LP used to evaluate the intracranial pressure (ICP), and the corresponding biochemical, and cytological examinations were all normal. The patient was then managed with the discontinuation of carboplatin along with the use of a high-dose systemic steroid, which was tapered off over a period of 10 weeks. After two years, optic atrophy and signs of optic nerve head ischemia were present in both eyes.^[4]^ Although they reported the case as papilledema, as the ICP was normal, it appears that it was in fact bilateral disc edema due to optic neuropathy.

In 2014, Lewis et al^[5]^ described a new onset blurry vision in the left eye of a 48-year-old female five days after completion of the fifth cycle of adjuvant carboplatin chemotherapy. All neurologic examinations were normal; however, on fluorescein angiography (FA), they found bilateral intense disc leakage in both eyes without demonstrating retinal vasculitis. This patient was also managed with the discontinuation of chemotherapy and prescription of the tapering usage of a moderate-dose systemic steroid. This case was unique since the right eye remained asymptomatic despite intense leakage revealed on FA. Five months after the initial presentation she developed optic atrophy in both eyes.^[5]^ Although they have described the condition as papilledema, as they did not perform LP and the computed tomography (CT) scan did not show any signs of elevated ICP, the term bilateral disc swelling may be more appropriate to describe the condition.

Our patient had bilateral disc edema secondary to carboplatin toxicity. The presentation and timing of optic nerve swelling in our patient were compatible with previous studies.^[4,5]^ We believe infiltrative and metastatic optic neuropathy is unlikely for two reasons. Firstly, LP was negative for malignant cells. Secondly, metastasis of squamous carcinoma of the tongue occurs through blood flow and the choroid is the tissue which is mostly involved. Additionally, involvement of both optic nerves at the same time after starting treatment with carboplatin, the progression of the disease during chemotherapy, and the stability after the discontinuation of chemotherapy make metastasis and infiltrative optic neuropathy unlikely. Vision remained stable after starting treatment with steroids; however, there was no improvement in the vision of the left eye despite intravenous, oral, and intravitreal corticosteroid therapy. MRI findings were also in favor of inflammatory process in the orbit which was previously reported in patients on systemic carboplatin therapy.^[2]^

Although paclitaxel-induced optic neuropathy has been previously reported in the literature,^[6,7,8]^ our patient is not a case of paclitaxel-induced optic neuropathy for the following six reasons. First, in contrast to inflammatory response and inflammatory optic neuropathy in carboplatin,^[3,4,5]^ the mechanism of paclitaxel ocular neurotoxicity mostly remains unclear since neither the ischemic (anatomical) nor electrophysiological (functional due to toxicity) hypotheses could fully explain the pathogenesis of paclitaxel ocular neurotoxicity in the previous studies.^[6,7]^ It is suggested that the visual symptoms and electrophysiological (electroretinography) changes observed after intravenous paclitaxel may be attributed to retinal vascular dysregulation. The occurrence of cystoid macular edema after paclitaxel treatment raises hypothesis of muller cell toxicity; however, abnormal visual-evoked potential closely resembles those observed in ischemic neuropathies.^[7]^ Nevertheless, the occurrence of photopsia in paclitaxel can be elucidated through the retinal toxicity theory which was not observed in our patient. In both case reports of paclitaxel optic neuropathy,^[6,7]^ a dilated fundoscopy exam was normal without swelling of the optic nerve head. Second, in the first two case reports,^[6,7]^ paclitaxel had been employed in combination with another medication (cisplatin or doxorubicin) and none of these studies specifically mentioned paclitaxel as the inciting agent for optic neuropathy. Third, paclitaxel causes peripheral neuropathy as its major side effect due to its effects on microtubule dynamics which can cause functional neuropathy. Presence of optic neuropathy along with optic nerve head swelling in the absence of peripheral neuropathy makes paclitaxel a less possible cause of optic neuropathy toxicity. Fourth, in paclitaxel patients,^[6,7,8]^ the visual acuity changes in the worst eye was significantly better than the visual acuity in our patient which makes the inflammatory process less possible in these cases. Additionally, positive visual phenomena such as photopsia which was not present in our patient may indicate involvement of retina in paclitaxel cases.^[6]^ Fifth, the inflammation of the optic nerves has not been demonstrated with the brain and orbital MRI in the patients with paclitaxel. Finally, the course of paclitaxel treatment had been completed 11 weeks before the patient's first presentation with progressive vision loss.

This patient was admitted to the oncology ward and the general hospital setting and the inaccessibility to a fundus camera, FA, and indocyanine green angiography posed as major limitations in the conduction of this research. Despite these limitations, this case report illustrates very important recommendation to oncologists and ophthalmologists.

Our findings in this case justify the need for the close monitoring of patients during their course of treatment with intravenous carboplatin therapy to discover pathology during the early stages of treatment and determine changes in visual acuity, visual field, and fundoscopy. Any subtle changes or vision complaint may rationalize the discontinuation of carboplatin in addition to starting systemic corticosteroids. These recommendations, however, need to be discussed with patients and the oncology team to ensure compatibility with the requirements of the case.

##  Financial Support and Sponsorship

None.

##  Conflicts of Interest

None.
